# *Scaffolds* Vasculares Descelularizados Provenientes de Vasos Sanguíneos de Placenta Bovina

**DOI:** 10.36660/abc.20220816

**Published:** 2023-06-01

**Authors:** Tarley Santos Oliveira, Igor Smirnow, Kadija Mohamed Santee, Maria Angelica Miglino, Rodrigo da Silva Nunes Barreto

**Affiliations:** 1 Departamento de Cirurgia Faculdade de Medicina Veterinária e Zootecnia Universidade de São Paulo São Paulo SP Brasil Departamento de Cirurgia, Faculdade de Medicina Veterinária e Zootecnia, Universidade de São Paulo, São Paulo, SP – Brasil

**Keywords:** Matriz Extracelular Descelularizada, Bioengenharia, Vasos Sanguíneos

## Abstract

**Fundamento:**

As doenças associadas ao aparelho circulatório são as principais causas de morbidade e mortalidade em todo o mundo, implicando a necessidade de implantes vasculares. Assim, a produção de biomateriais vasculares tem se mostrado uma alternativa promissora às terapias utilizadas em estudos e pesquisas relacionados à fisiologia vascular.

**Objetivos:**

O presente projeto visa ao desenvolvimento artificial de vasos sanguíneos pela recelularização de *scaffolds* vasculares derivados de vasos placentários bovinos.

**Métodos:**

A superfície corioalantoide da placenta bovina foi utilizada para produzir biomateriais descelularizados. Para a recelularização, 2,5 x 10^4^ células endoteliais foram semeadas acima de cada fragmento de vaso descelularizado durante três ou sete dias, quando a cultura foi interrompida e os fragmentos foram fixados para análise de adesão celular. Biomateriais descelularizados e recelularizados foram avaliados por histologia básica, microscopia eletrônica de varredura e imuno-histoquímica.

**Resultados:**

o processo de descelularização produziu vasos que mantiveram a estrutura natural e o conteúdo de elastina, e não foram observadas células e gDNA remanescentes. Além disso, células precursoras endoteliais se ligaram ao lúmen e à superfície externa do vaso descelularizado.

**Conclusão:**

nossos resultados mostram a possibilidade de usos futuros desse biomaterial na medicina cardiovascular, como, por exemplo, no desenvolvimento de vasos artificiais.

## Introdução

As doenças crônicas não transmissíveis (DCNT) têm aumentado principalmente devido ao sedentarismo, alimentação industrializada, ingestão calórica alta, consumo de álcool e tabagismo. Dentre as DCNT, as relacionadas à insuficiência do sistema circulatório são as principais causas de morbidade e mortalidade mundial.^1-3^ Essa insuficiência é majoritariamente tratada por cirurgia vascular e são necessários vasos viáveis, que geralmente são derivados da dissecção de vasos periféricos autólogos e implantação no local afetado. No entanto, são comumente descritas complicações infecciosas, necrose e deiscência no local da retirada dos vasos. Em alguns casos, coloca-se um stent no vaso lesionado em vez do fragmento do vaso periférico, porém possíveis complicações são fraturas e hiperplasia intimal.^4,5^

Nesse cenário, avanços significativos foram alcançados pela bioengenharia na produção de novos órgãos funcionais ou funcionalizados, como o desenvolvimento de vasos sanguíneos projetados para dar suporte à vascularização funcional.^6-8^ Até agora, vasos de pequena escala foram produzidos e a maioria deles é formada a partir de derivados de polímeros sintéticos rígidos.^6,9^ Por outro lado, vasos projetados que mantenham sua função estrutural e celular por períodos longos ainda não foram produzidos.

Assim, a produção de biomateriais vasculares por recelularização, e especialmente por bioimpressão, tem se mostrado uma alternativa promissora às terapias utilizadas em doenças e estudos relacionados à fisiologia vascular.^9,10^ Nosso objetivo aqui é produzir biomateriais vasculares derivados de vasos placentários bovinos descelularizados que possam ser usados como sua estrutura tridimensional natural ou como biogéis.

## Materiais e Métodos

### Amostra e células

Para isolamento dos vasos, placenta bovina com idade estimada de 270 dias de gestação foi obtida em abatedouro. Para os ensaios de citocompatibilidade, duas células progenitoras endoteliais foram utilizadas: saco vitelino canino (SV) e células do saco vitelino canino com superexpressão do fator de crescimento endotelial vascular (VEGF) e da proteína verde fluorescente (eGFP) (SV-VEGF).^11^ Todos os experimentos foram aprovados pelo Comitê de Ética no Uso de Animais da Faculdade de Medicina Veterinária e Zootecnia da Universidade de São Paulo, sob o protocolo número 9715100718.

### Protocolo de descelularização

A superfície corioalantóide da placenta bovina foi utilizada para produzir biomateriais descelularizados. O corioalantoide foi individualizado e as artérias umbilicais foram canuladas com cateteres nº 14 e conectadas ao biorreator ORCA (Harvard Aparattus, EUA). Inicialmente, a perfusão foi realizada com solução tampão de fosfato (PBS: 136,9 mM NaCl, 26,8 mM KCl, 14,7 MM KH2PO4 e 8,1 mM Na2HPO4.7H2O; pH 7,2) com volume constante de 0,5 ml/min até completa limpeza do sistema vascular, aproximadamente 24 horas. Em seguida, uma solução de dodecil sulfato de sódio a 0,01% (SDS, Sigma-Aldrich 11767289001) foi perfundida em água destilada também a 0,5ml/min por 24 horas. Posteriormente, a solução foi alterada para SDS 0,1% por dois dias, para 0,25% por um dia, para 0,5% por 1 dia e para 1,0% por um dia. O corioalantoide descelularizado foi então perfundido com Triton X-100 a 1% (#0694-1L, Amresco-Solon, EUA) por três horas. Por fim, foi lavado com PBS por 24 horas, perfazendo um total de 10 dias.

Após a descelularização, todo o sistema vascular da superfície alantocoriônica foi isolado do cotilédone e da membrana. Em seguida, os vasos foram preservados em paraformaldeído (PFA) a 4% para validação da descelularização ou congelados rapidamente para ensaio de citocompatibilidade e produção de hidrogel.

### Validação da descelularização

Para validação da descelularização por análise histológica, as amostras preservadas em PFA foram rotineiramente desidratadas, diafanizadas e embebidas em parafina. Em seguida, foram seccionadas em micrótomo manual (Leica RM2125 RT) em fatias de 5 µm de espessura e transferidas para lâminas histológicas. Para verificar a ausência de núcleos celulares visíveis, as lâminas foram coradas em hematoxilina e eosina (HE) ou 4’,6’-diamino-2-fenil-indol (DAPI) e observadas em microscópio Nikon Eclipse 80l sob luz ou epifluorescência, respectivamente.

Além disso, a validação da descelularização foi realizada pela quantificação do ácido desoxirribonucleico genômico remanescente (gDNA) que foi extraído por precipitação de sal, adaptado de Olerup e Zetterquist,^12^ conforme descrito por Barreto et al.^13^

### Ensaio de recelularização

Primeiramente, fragmentos de vasos descelularizados de 4 cm foram esterilizados por lavagem com suplemento de PBS com 2% de antibióticos (penicilina e estreptomicina), seguido de banho de álcool 70% e luz ultravioleta (UV).

Para a recelularização, 2,5 x 10^4^ células SV ou 2,5 x 10^4^ células SV-VEGF foram semeadas acima de cada fragmento de vaso descelularizado durante três ou sete dias, quando a cultura foi interrompida e os fragmentos foram fixados para análise de adesão celular. A cultura foi realizada com meio alfa mínimo essencial (α-MEM) (LGC Biotechnology), suplementado com 10% de soro fetal bovino e 1% de estreptomicina/penicilina, sob temperatura de 37 °C e 5% de CO_2_.

### Validação da recelularização

Para verificar a adesão celular, alguns fragmentos de vasos recelularizados foram corados com DAPI e observados sob microscópio confocal a laser (Olympus Fluo View 1000 - FV1000). Os demais fragmentos foram fixados em Karnovsky (4% PFA e 2,5% glutaraldeído em tampão cacodilato de sódio 0,1 M pH 7,2) e posteriormente fixados em tetróxido de ósmio a 1% (SEM R 148- HATFIELD, EUA) por 90 minutos. Eles foram desidratados em uma série crescente de etanol sob agitação, depois passaram por um processo de secagem automatizado (MSCPD 300, Leica) e foram metalizados com ouro (#K550, Emitech-Ashford, Reino Unido). Também foi realizada imuno-histoquímica, consequentemente,^13^ para verificar a elastina na parede do vaso e VEGF da linha celular SV-VEGF.

## Resultados

**Figura 1:**
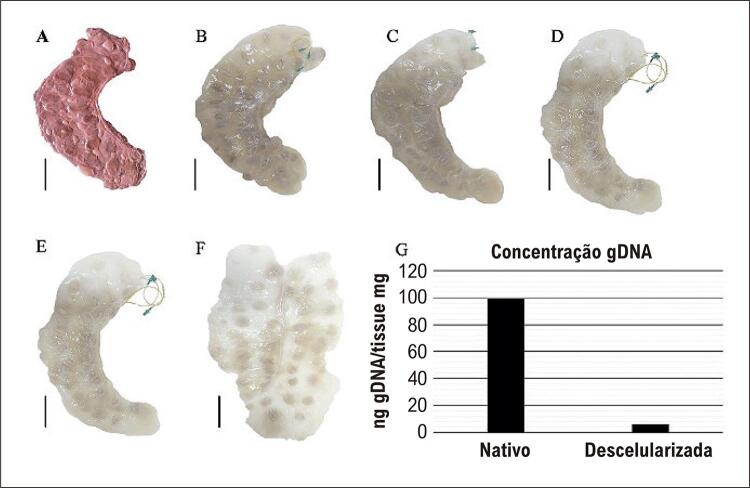
– Descelularização dos vasos placentários bovinos: A: bar = 10 cm, placenta dissecada e artéria e veia umbilical canuladas. B: bar = 10 cm, início do processo de descelularização. C-E: bar = 10 cm, avanço da descelularização. F: bar = 10 cm, conclusão da descelularização, com manutenção da estrutura. G: ácido desoxirribonucleico genômico (gDNA) nos scaffolds vasculares da superfície corioalantoide da placenta bovina descelularizada.

Após a descelularização, não foram observados núcleos visíveis nos fragmentos dos vasos nem pela coloração por hematoxilina e eosina (HE) nem pela coloração por DAPI (Figura Central – D2). Além disso, foi quantificado menos de 50 ng de gDNA por mg de vaso descelularizado (Figura 1). Com relação à conservação do arranjo vascular dos vasos da placenta bovina, observou-se, mesmo em vasos de até 5 mm de diâmetro (Figura 1-F), pela microscopia eletrônica de varredura, que os vasos descelularizados apresentavam manutenção estrutural e lúmen poroso (Figura Central – A4).

**Figura Central:**
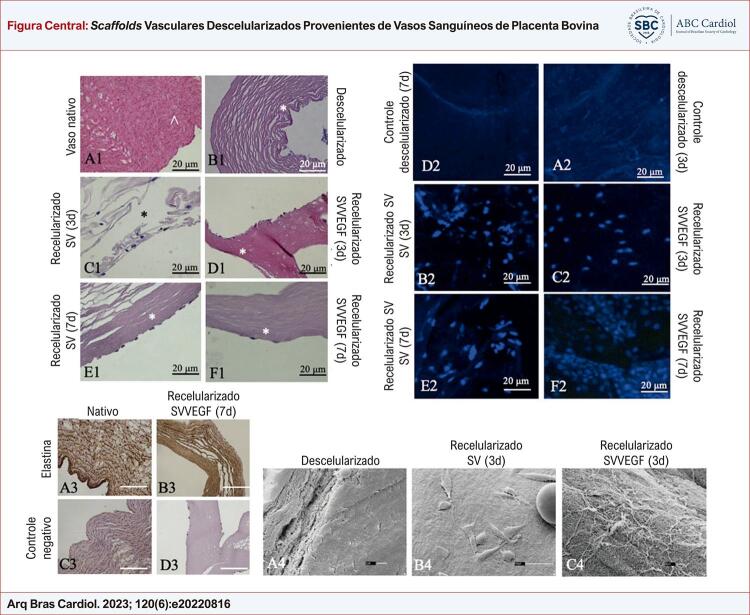
: *Scaffolds* Vasculares Descelularizados Provenientes de Vasos Sanguíneos de Placenta Bovina

Análise de vasos de placenta bovina descelularizada. Hematoxilina eosina, (A1) Vaso nativo, Bar= 20 μm, (^) lúmen: fibras da matriz extracelular coradas em rosa e núcleo celular em roxo; (B1) Descelularizado, Bar= 20 μm, (*) superfície externa: núcleos celulares ausentes; (C-F1) Recelularizado, Bar= 20 μm, (*) superfície externa: (C1), Recelularizado três dias (linha SV), núcleos celulares corados em roxo aderidos à superfície externa do biomaterial; (D1), Recelularizado três dias com células do saco vitelino canino com superexpressão de fator de crescimento endotelial vascular (VEGF) e proteína verde fluorescente aumentada (eGFP) (linhagem SV-VEGF), fibras da matriz extracelular coradas em rosa e núcleos celulares em roxo aderidos à superfície externa do biomaterial; (E1), Células do saco vitelino caninas recelularizadas com sete dias (linhagem SV), núcleos celulares corados em roxo aderidos à superfície externa do biomaterial; (F1), Recelularizado sete dias com células (linhagem SV-VEGF), fibras da matriz extracelular coradas em rosa, núcleos celulares em roxo aderidos à superfície externa do biomaterial. (A4) bar = 2 μm, descelularizado: Observe a preservação das fibras da matriz extracelular do biomaterial vascular; Recelularização com três dias de cultura (B4, C4): (B4) bar = 30 μm, com células da VS aderidas à parede do biomaterial vascular; (C4) bar = 10 μm, células SV-VEGF aderidas à parede do biomaterial vascular. Ensaio de imuno-histoquímica: expressão de elastina no biomaterial vascular da superfície corioalantoide bovina nativa e recelularizada com células SV-VEGF. Legenda: Bar=20 µm, (A3) Vaso sanguíneo nativo da superfície corioalantoide da placenta bovina, observe a expressão de elastina em toda a parede vascular; (B3) Vaso sanguíneo nativo da superfície corioalantoide da placenta bovina, controle negativo corado com hematoxilina; (C3) Recelularização de 7 dias de biomaterial vascular placentário bovino com células SV-VEGF, observando a expressão de elastina em toda a parede vascular; (D3) Recelularização de 7 dias de biomaterial vascular placentário bovino com células SV-VEGF, controle negativo corado com hematoxilina. Ensaio de recelularização: Barra 20 µm; (A2-C2) 3 dias de cultivo; (A2) Biomaterial vascular controle descelularizado, observe a ausência de núcleos; (B2) Biomaterial recelularizado com células progenitoras endoteliais (SV) do saco vitelino canino, observe núcleos corados em azul por 4’,6’-diamino-2-fenil-indol (DAPI); (C2) Biomaterial recelularizado com células SV-VEGF, observe núcleos corados em azul por DAPI, eGFP não observada; (D2-F2) 7 dias de cultivo; (D2) controle descelularizado de biomaterial vascular, observe a ausência de núcleos; (E2) Biomaterial recelularizado com células SV, observe núcleos corados em azul por DAPI; (F2) Biomaterial recelularizado com células SV-VEGF, observe núcleos corados em azul por DAPI.

Após a recelularização, mesmo no terceiro dia de cultura, foi possível observar células na superfície e no lúmen do vaso descelularizado; entretanto, visualmente, o número de células aumentou no sétimo dia. Além disso, as células SV-VEGF foram mais numerosas do que as células SV, tanto no terceiro quanto no sétimo dia. Essas células foram observadas pelas colorações HE e DAPI (Figura Central – D1 e F2).

O padrão de distribuição da elastina foi semelhante nos vasos placentários nativos, descelularizados e recelularizados (Figura Central – A3, B3 e D3).

## Discussão

A placenta bovina é um órgão que, após a descelularização, pode ser utilizado como fonte de biomaterial, principalmente *scaffolds* vasculares. Mesmo após o processo de descelularização, tanto os cotilédones^13^ quanto a membrana corioalantoide^14^ mantiveram sua estrutura e composição da matriz extracelular preservadas. Aqui produzimos vasos descelularizados com estrutura e manutenção da elastina, mesmo em vasos de pequeno diâmetro. Além disso, esses vasos descelularizados apresentavam citocompatibilidade com células precursoras endoteliais (células SV e SV-VEGF) e os resultados morfológicos e comportamentais dessas linhagens celulares já haviam sido descritos e permanecem de acordo com os apresentados pela recelularização em outros biomateriais, conforme demonstrado por Fratini et al.^15^

Ademais, outra alternativa para o uso desse vaso descelularizado é sua digestão para produzir biogéis ricos em colágeno, que podem ser usados para produzir vasos de bioengenharia com diâmetros e tamanhos diferentes.^6,16,17^

## Conclusão

Os vasos da placenta bovina podem produzir biomateriais descelularizados viáveis e citocompatíveis que podem ser uma fonte de biomateriais vasculares estruturalmente naturais, bem como perspectivas futuras para vasos de bioengenharia.
