# Supplementary material for: Scaffolds Vasculares Descelularizados Provenientes de Vasos Sanguíneos de Placenta Bovina
Source: Arq Bras Cardiol. 2023 Jun 1;120(6):e20220816. [Article in Portuguese] doi: 10.36660/abc.20220816 (PMC10263409; doi:10.36660/abc.20220816)
Supplement: Translation

# Translation: Decellularized Vascular Scaffolds Derived from Bovine Placenta Blood Vessels

**Authors:** Tarley Santos Oliveira, Igor Smirnow, Kadija Mohamed Santee, Maria Angelica Miglino, Rodrigo da Silva Nunes Barreto

**Affiliations:** 1 Brasil Departamento de Cirurgia, Faculdade de Medicina Veterinária e Zootecnia, Universidade de São Paulo, São Paulo, SP – Brasil

**Keywords:** Decellularized Extracellular Matrix, Bioengineering, Blood Vessels

## Abstract

Diseases associated with the circulatory system are the main causes of worldwide morbidity and mortality, implying the need for vascular implants. Thus, the production of vascular biomaterials has proven to be a promising alternative to therapies used in studies and research related to vascular physiology.

**Objectives:**

The present project aims to achieve the artificial development of blood vessels through the recellularization of vascular scaffolds derived from bovine placental vessels.

**Methods:**

The chorioallantoic surface of the bovine placenta was used to produce decellularized biomaterials. For recellularization, 2.5 x 10^4^ endothelial cells were seeded above each decellularized vessel fragment during three or seven days, when culture were interrupted, and the fragments were fixed for cell attachment analysis. Decellularized and recellularized biomaterials were evaluated by basic histology, scanning electron microscopy, and immunohistochemistry.

**Results:**

The decellularization process produced vessels that maintained natural structure and elastin content, and no cells or gDNA remains were observed. Endothelial precursor cells were also attached to lumen and external surface of the decellularized vessel.Conclusion: Our results show a possibility of future uses of this biomaterial in cardiovascular medicine, as in the development of engineered vessels.

## Introduction

Noncommunicable diseases (NCDs) have been increased mainly due to a sedentary lifestyle, industrial food, high-calorie intake, alcohol, and tobacco consumption. Among NCDs, those related to the circulatory system’s failure are the main causes of worldwide morbidity and mortality.^1^ This failure is mainly treated by vascular surgery and viable vessels are needed, which are generally derived from autologous peripheral vessel dissection and implantation in the affected site. However, commonly infectious complications, necrosis, and dehiscence are described at the vessel removal site. In some cases, a stent is placed in the injured vessel instead of a peripheral vessel fragment; however, possible complications include fractures and intimal hyperplasia.^4,5^

In this scenario, significant advances have been made by bioengineering in the production of new functional or functionalized organs, such as engineered blood vessel development to support functional vascularization.^6-8^ So far, small-scale vessels have been produced, most of which are formed from derivatives of rigid synthetic polymers.^6,9^ Otherwise, engineered vessels that maintain their structural and cellular function for long periods have not yet been produced.

Thus, the development of vascular biomaterials by recellularization, and especially by bioprinting, have proven to be promising alternatives to support therapies used in diseases and studies related to vascular physiology.^9,10^ The present study thus aimed to produce vascular biomaterials derived from decellularized bovine placental vessels that could be used as its natural tridimensional structure or as biogels.

## Materials and Methods

### Sample and cells

For vessel isolation, bovine placenta, with an estimated age of 270 days of pregnancy, was obtained at a slaughterhouse. For cytocompatibility assays, two endothelial progenitor cells were used: canine yolk sac (SV) and canine yolk sac cells with vascular endothelial growth factor (VEGF) and enhanced green fluorescent protein (eGFP) overexpression (SV-VEGF).^11^ All experiments were approved by the Committee on Ethics in the Use of Animals of the Faculty of Veterinary Medicine and Animal Science at the University of São Paulo, logged under protocol number 9715100718.

### Decellularization protocol

The chorioallantoic surface of the bovine placenta was used to produce decellularized biomaterials. The chorioallantois was individualized and the umbilical arteries cannulated with #14 catheters and attached to the ORCA bioreactor (Harvard Aparattus, USA). Initially, perfusion was performed with a phosphate buffer solution (PBS: 136.9 mM NaCl, 26.8 mM KCl, 14.7 MM KH2PO4, and 8.1 mM Na2HPO4.7H2O; pH 7.2) with a constant volume of 0.5 ml/min until complete cleaning of the vascular system, approximately 24 hours. A 0.01% solution of sodium dodecyl sulfate (SDS, Sigma-Aldrich 11767289001) was then perfused in distilled water, also under 0.5ml/min for 24 hours. Subsequently the solution was changed to 0.1% SDS for two days, to 0.25% for one day, to 0.5% for 1 day, and to 1.0% for one day, respectively. The decellularized chorioallantois was then perfused with 1% Triton X-100 (#0694-1L, Amresco-Solon, USA) for three hours. Finally, it was washed with PBS for 24 hours, making a total of 10 days.

After decellularization, the entire vascular system from the allantochorionic surface was isolated from the cotyledon and the membrane. The vessels were then preserved in 4% paraformaldehyde (PFA) for decellularization validation or snap frozen for cytocompatibility assay and hydrogel production.

### Decellularization validation

For decellularization validation by histological analysis, the PFA preserved samples were routinely dehydrated, diaphanized, and paraffin embedded. These were then sectioned using a manual microtome (Leica RM2125 RT) into 5 µm thick slices and transferred to histological slides. To verify the absence of visible cell nuclei, the slides were stained in Hematoxylin and Eosin (H&E) or 4’,6’-diamino-2-fenil-indol (DAPI) stained and observed under a Nikon Eclipse 80l microscope under light or epifluorescence, respectively.

The decellularization validation was also performed by quantifying the remaining genomic deoxyribonucleic acid (gDNA), which was extracted by salt precipitation, adapted from Olerup and Zetterquist,^12^ as described by Barreto et al.^13^

### Recellularization assay

First, decellularized vessel fragments of 4 cm were sterilized by washing with PBS supplement with 2% antibiotics (penicillin and streptomycin), followed by a 70% alcohol bath and ultraviolet (UV) light.

For recellularization, 2.5 x 10^4^ SV cells or 2.5 x 10^4^ SV-VEGF cells were seeded above each decellularized vessel fragment for three or seven days, when the cultures were interrupted and the fragments were fixed for cell attachment analysis. Culture was performed with alpha minimum essential medium (α-MEM) (LGC Biotechnology), supplemented with 10% fetal bovine serum and 1% streptomycin/penicillin antibiotic, under 37°C and 5% CO_2_.

### Recellularization validation

To verify cell attachment, some recellularized vessel fragments were DAPI stained and observed under laser confocal microscope (Olympus Fluo View 1000 - FV1000). Other fragments were fixed in Karnovsky (4% PFA and 2.5% glutaraldehyde in 0.1 M sodium cacodylate buffer at pH 7.2) and then post-fixed in 1% osmium tetroxide (SEM R 148- HATFIELD, USA) for 90 minutes. These were then dehydrated in an increasing series of Ethanol under agitation, passed through an automated drying process (MSCPD 300, Leica), and metallized with gold (#K550, Emitech-Ashford, UK). Immunohistochemistry was also performed^13^ to verify elastin on the vessel wall and VEGF from the SV-VEGF cell line.

## Results

After decellularization, no visible nuclei were observed in the vessel fragments, neither by hematoxylin and eosin (HE) nor by DAPI stains (Central Illustration - D2). Moreover, less than 50 ng of gDNA per mg of the decellularized vessel was quantified (Figure 1). Regarding the conservation of the vascular arrangement of the bovine placenta vessels, it was observed, even in vessels up to 5 mm in diameter (Figure 1-F), viewed through scanning electron microscopy, that the decellularized vessels showed structural maintenance and a porous lumen (Central Illustration – A4).

**Central Illustration:** : Decellularized Vascular Scaffolds Derived from Bovine Placenta Blood Vessels

Analysis of decellularized bovine placental vessel. Hematoxylin eosin, (A1) Native vessel, Bar= 20 μm, (^) lumen: extracellular matrix fibers stained in pink and cell nucleus in purple; (B1) Decellularized, Bar= 20 μm,(*) external surface: absent cell nuclei; (C-F1) Recellularized, Bar= 20 μm, (*) external surface: (C1), Recellularized three days (SV line), cell nuclei stained in purple adhered to the external surface of the biomaterial; (D1), Recellularized three days with canine yolk sac cells with vascular endothelial growth factor (VEGF) and enhanced green fluorescent protein (eGFP) overexpression (SV-VEGF lineage), extracellular matrix fibers stained in pink and cell nuclei in purple adhered to the external surface of the biomaterial; (E1), Recellularized seven days canine yolk sac cells (SV-lineage), cell nuclei stained in purple adhered to the external surface of the biomaterial; (F1), Recellularized seven days with cells (SV-VEGF lineage), extracellular matrix fibers stained in pink, cell nuclei in purple adhered to the external surface of the biomaterial. (A4) bar = 2 μm, decellularized: Observe preservation of fibers of the extracellular matrix of the vascular biomaterial; Recellularization with three days of culture (B4, C4): (B4) bar = 30 μm, with SV cells adhered to the vascular biomaterial wall; (C4) bar = 10 μm, SV-VEGF cells adhered to the vascular biomaterial wall. Immunohistochemistry assay: elastin expression in the vascular biomaterial of the native bovine chorioallantoic surface, and recellularized with SV-VEGF cells. Caption: Bar=20 µm, (A3) native blood vessel of the chorioallantoic surface of the bovine placenta, observe elastin expression in the entire vascular wall; (B3) native blood vessel from the chorioallantoic surface of the bovine placenta, negative control stained with hematoxylin; (C3) 7-day recellularization of bovine placental vascular biomaterial with SV-VEGF cells, observing elastin expression in the entire vascular wall; (D3) 7-day recellularization of bovine placental vascular biomaterial with SV-VEGF cells, negative control stained with hematoxylin. Recellularizarion assay: Bar 20 µm ; (A2-C2) 3 days of cultivation; (A2) decellularized control vascular biomaterial, observe absent nuclei; (B2) biomaterial recellularized with canine yolk sac endothelial progenitor cells (SV), observe nuclei stained in blue by 4’,6’-diamino-2-fenil-indol (DAPI); (C2) recellularized biomaterial with SV-VEGF cells, observe nuclei stained blue by DAPI, eGFP not observed; (D2-F2) 7 days of cultivation; (D2) vascular biomaterial decellularized control, observe absent nuclei; (E2) biomaterial recellularized with SV cells, observe nuclei stained in blue by DAPI; (F2) Biomaterial recellularized with SV-VEGF cells, observe nuclei stained in blue by DAPI.

**Figure 1:** – Decellularization of the bovine placenta vessels: A: bar = 10 cm, placenta dissected, and umbilical artery and vein cannulated. B: bar = 10 cm, beginning of the decellularization process. C-E: bar = 10 cm, progression of decellularization. F: bar = 10 cm, completion of decellularization, with maintenance of the structure. G: genomic deoxyribonucleic acid (gDNA) in the vascular scaffolds of the chorioallantoic surface of the decellularized bovine placenta.

After recellularization, even on day three of the culture, it was possible to observe the cells on the decellularized vessel surface and lumen; however, visually, the number of cells increased on day seven. The SV-VEGF cells were also more numerous than the SV cells, both on day three and seven. Those cells were observed both by HE and DAPI stains (Central Illustration – D1 and F2).

The elastin distribution pattern was similar in native, decellularized, and recellularized placental vessels (Central Illustration – A3, B3 and D3).

## Discussion

Bovine placenta is an organ that, after decellularization, can be used as a source of biomaterial, especially vascular scaffolds. Even after the decellularization process, both the cotyledons,^13^ as well as the chorioallantoic membrane,^14^ maintained their structure and extracellular matrix composition preserved. Here, we produced decellularized vessels with structure and elastin maintenance, even in small diameter vessels. Those decellularized vessels had cytocompatibility with endothelial precursor cells (SV and SV-VEGF cells), and the morphological and behavioral results of these cell lines had already been described and remain in accordance with those presented by recellularization in other biomaterials, as demonstrated by Fratini et al.^15^

Furthermore, another alternative to use this decellularized vessel is its digestion to produce biogels that are rich in collagen and can be used to produce bioengineered vessels with different diameters and sizes.^6,16,17^

## Conclusion

The bovine placenta vessels can produce viable and cytocompatible decellularized biomaterials that can be a source of structurally natural vascular biomaterials, as well as future prospects for bioengineered vessels.
